# Editorial: Contemporary Management of Intracranial Metastatic Disease

**DOI:** 10.3389/fonc.2019.00818

**Published:** 2019-08-28

**Authors:** Sunit Das, Arjun Sahgal

**Affiliations:** ^1^Li Ka Shing Knowledge Institute, St. Michael's Hospital, Toronto, ON, Canada; ^2^Division of Neurosurgery, University of Toronto, Toronto, ON, Canada; ^3^Department of Radiation Oncology, Sunnybrook Hospital, Toronto, ON, Canada

**Keywords:** brain metastases (BM), surgery, immunotherapy, focused ultrasound, chemotherapy, targeted therapy, radiotherapy, SRS

On behalf of my radiation oncology partner, Dr. Arjun Sahgal, I am pleased to offer to the readers of *Frontiers in Oncology* our Research Topic entitled, “Contemporary management of intracranial metastatic disease.” The development of intracranial metastatic disease (IMD) complicates the course of ~20% of patients with cancer, with the highest frequency of brain metastases arising in patients with melanoma, breast cancer, and lung cancer (1, 2). Modern therapeutic options available for treatment of IMD include surgical resection, stereotactic radiosurgery (SRS) and to an expanding degree, targeted, and immuno-therapies (3). Despite improvements in patient survival with more aggressive treatment options as compared to the prior standard of palliative whole brain radiation, outcomes for patients who develop IMD remain dispiriting. There is need to celebrate our advances; but a major collaborative multidisciplinary effort is needed to push the field to achieve more meaningful survival benefits for our patients with IMD.

In this Research Topic, we have assembled work detailing the latest innovations in brain metastases imaging and management. Mehrabian et al. have reviewed work in the MR imaging field to identify advanced biomarkers that characterize the cellular, biophysical, micro-structural, and metabolic features of tumors to improve the management of brain metastases from early detection and diagnosis, to evaluating treatment response. Venur et al. review the contribution of genomic analysis of brain metastases to our understanding of variations in the driver mutations compared to the primary malignancy, and provide an in-depth review of the completed and ongoing clinical trials of drugs targeting the molecular pathways enriched in brain metastases. Kamath and Kumthekar review the biological rationale for systemic immunotherapy to treat CNS metastatic disease, and summarize existing clinical data on immune checkpoint inhibitors in this setting and ongoing clinical trials designed to study immune checkpoint inhibitor therapy in patients with IMD. Meng et al. review the prospect of focused ultrasound-mediated blood-brain barrier disruption to improve local drug delivery for patients with IMD. Minimally invasive surgical strategies using tubular retractors or laser interstitial thermal therapy (LITT) have been presented by Marenco-Hillembrand et al. and Salehi et al., respectively. Routman et al. review data to support the use of SRS as a neoadjuvant therapy to improve treatment contouring and diminish the risk of tumor cell dissemination during surgical resection and associated leptomeningeal spread. Masucci and Routman et al. detail the role of hyprofractionated SRS and post-operative SRS, respectively, for patients with IMD. Robin and Rusthoven and Schwendner et al. have reviewed efforts to minimize disease- and treatment-related cognitive and motor decline in patients with IMD. Vellayappan et al. explore the pathophysiology of radiation necrosis, risk factors for its development, and the strategies for its evaluation and management. Finally, Samuel Chao and colleagues define a treatment algorithm to guide treatment of patients with a limited number of brain metastases, while Roberge et al. describe plans for a prospective randomized control trial to define the role of SRS in patients with more severe burden of IMD.

In all of this, we owe thanks to our many colleagues who contributed to this Research Topic and offered us their time, intellect and energy. Many thanks as well to Jakob Mainusch and the editorial team at *Frontiers*. We thank Frontiers for appreciating the importance of this topic and for supporting the collection. We hope that you find it a valuable resource.

## Author Contributions

SD wrote and edited the manuscript. AS edited the manuscript and provided guidance on its writing.

### Conflict of Interest Statement

The authors declare that the research was conducted in the absence of any commercial or financial relationships that could be construed as a potential conflict of interest.
